# From Daily Life Processes to Long-Term Health: Innovations in Measurement-Burst Designs

**DOI:** 10.1093/geroni/igaf122.822

**Published:** 2025-12-31

**Authors:** Sun Ah Lee, David Almeida

## Abstract

Measurement-burst designs offer a nuanced approach for examining developmental processes by capturing both short-term dynamics and long-term changes in psychosocial experiences, behaviors, and health. Despite their unique capacity to assess changes across multiple temporal intervals, these designs remain underutilized, due in part to limited refinement of their methodological application. This symposium highlights innovative research questions and modeling approaches that leverage measurement-burst data to examine developmental changes in daily or momentary experiences and their implications for health and well-being across adulthood. Lee and Almeida use two bursts of daily diary and biomarker data to investigate the mediating role of daily affect dynamics in the longitudinal and bidirectional relationship between depressive symptoms and inflammation. Rush et al. use three bursts of daily diary data to examine longitudinal changes in daily stress reactivity over 20 years and its mediating role in linking declines in functional health and life satisfaction. Chai et al. use three bursts of ecological momentary assessments to evaluate the impact of a cognitive training intervention on longitudinal changes in daily subjective sleep and age. Witzel et al. use two bursts of daily diary data to explore age differences and longitudinal changes in the daily associations between positive experiences and physical symptoms. Almeida will discuss recent innovations and opportunities in measurement-burst designs and their potential to advance research on daily experiences and health across adulthood. This symposium demonstrates how measurement-burst approaches enhance our understanding of developmental changes across different timescales and provide insights into healthy aging. Measurement, Statistics, and Research Design Interest Group Sponsored Symposium

